# Prevalence and correlates of concurrent use of cigarettes, electronic cigarettes, and waterpipes among Serbian youth

**DOI:** 10.18332/tid/111357

**Published:** 2019-09-13

**Authors:** Biljana Kilibarda, Dejana Vukovic, Srmena Krstev

**Affiliations:** 1 Institute of Public Health of Serbia, Belgrade, Serbia; 2 Institute of Social Medicine, Faculty of Medicine, University of Belgrade, Belgrade, Serbia; 3 Association ‘Health Mission’, Belgrade, Serbia

**Keywords:** Serbia, youth, e-cigarettes, concurrent tobacco use, waterpipes

## Abstract

**INTRODUCTION:**

Concurrent use of tobacco products is associated with an increased risk of nicotine dependence and smoking-related health complications. Growing popularity of concurrent use of cigarettes and electronic cigarettes and/or waterpipe tobacco is of concern, especially due to the adolescents’ exposure to nicotine and call for the better understanding of patterns and predictors of multiple product use.

**METHODS:**

This is a secondary analysis of cross-sectional data obtained through the 2017 Global Youth Tobacco Survey conducted in Serbia on a nationally representative sample of 3362 students aged 13–15 years. Students were categorized into eight groups based on their experience with cigarette, e-cigarette and waterpipe use. To explore differences in sociodemographic and psychosocial characteristics, students were further placed in four groups: non-users; exclusive cigarette users; users of e-cigarette and/or waterpipe who do not smoke cigarettes; cigarette and other product users.

**RESULTS:**

We show that among the 13–15 years old students, the most common pattern of tobacco/nicotine use is waterpipe and/or e-cigarette use with no cigarette smoking (7.5%, 95% CI: 6.6–8.4) followed by exclusive cigarette use (5.8 %, 95% CI: 5.0–6.6). Among cigarette smokers, 52.8% were exclusive cigarette smokers. Having the majority of their friends smoking is a mutual predictor for exclusive cigarette (AOR=33.2, 95% CI: 14.52–75.90) waterpipe and/or e-cigarette (AOR=2.57, 95% CI: 1.56–4.25) and cigarette and other products use (AOR=52.3, 95% CI:12.28–223.22) compared to no use of any product, and the same in the case of exposure at the point-of-sale marketing: exclusive cigarette vs not any product users (AOR=1.82, 95% CI: 1.22–2.73); waterpipe and or/e-cigarette vs not any product users (AOR=1.64, 95% CI:1.18–2.28); and cigarette and other products vs not any product users (AOR=3.40, 95% CI: 1.99–5.80).

**CONCLUSIONS:**

Tobacco control interventions should address dual- and poly-tobacco use with special focus on inter-personal factors and protection from exposure to advertising of e-cigarettes and waterpipes.

## INTRODUCTION

Tobacco control measures that are implemented worldwide, to a different extent and with different impact, have resulted in a decrease in cigarette smoking among adolescents. In parallel with the decline in the prevalence of cigarette smoking among youth in Europe^1^ and the US^2^, data from different studies demonstrate the growing popularity of waterpipe tobacco smoking^3^ and e-cigarette use^4^, worldwide.

E-cigarette use and waterpipe tobacco smoking are of growing concern due to the adolescents’ exposure to nicotine. There is often a misconception that nicotine/tobacco dependence cannot be developed by waterpipe tobacco smoking^5^. Furthermore, nicotine exposure and nicotine dependence specific to e-cigarettes among adolescents are severe public health issues^6^.

An additional challenge is the popularity of dual-use of cigarettes and electronic cigarettes^7^ and/or waterpipe tobacco, for which a better understanding is of great importance^8^.

Available research provides evidence that dual- and poly-tobacco use represents a significant public health problem^9^. Also, concurrent use of tobacco products is associated with increased risk of nicotine dependence and smoking-related health complications^10^. Due to changing patterns of tobacco/nicotine use, it is necessary to have data on prevalence and patterns of concurrent use of these products. Otherwise, data that show a decrease in smoking might mask the problem of exposure to nicotine caused by e-cigarette use, waterpipe tobacco smoking and other emerging tobacco products.

The majority of studies on concurrent use of tobacco/nicotine products are from the US^2,8,11^ and some highly populated countries^12^ and they often face different challenges compared to those in other regions of the world. Limited data are available for Europe, mostly coming from EU member states^13,14^. Data on dual-tobacco/nicotine use among youth coming from developing European countries that are not members of the EU are scarce, and to our knowledge, none of the studies explored concurrent use of cigarettes, electronic cigarettes, and waterpipe. The Western Balkan region in Europe is especially vulnerable as these countries differ in legislation from the EU member states where the Tobacco Product Directive (2014/40/EU) has been implemented and regulates both tobacco products (e.g. packaging, labelling, ingredients) and electronic cigarettes. In Serbia, the most populated country of this region, the regulation of tobacco products and e-cigarettes is neither in compliance with EU legislation and recommendations nor with the provisions of the FCTC^15^. Also, in Serbia, there are four tobacco companies, something which is already a challenge.

Apart from the need for understanding the patterns of tobacco/nicotine use, research worldwide requests a better understanding of predictors of dual- and poly-tobacco use patterns in youth^16^. Several factors are known to influence the initiation and the continuity of cigarette smoking and tobacco use. Apart from inter- and intra-personal factors (personality, social support and socioeconomic status, self-esteem) and economic factors (especially the price of tobacco), many studies confirm the importance of environmental factors such as social factors (peers, siblings, parents) and the exposure to tobacco advertising, promotion, and sponsorship^17,18^.

Therefore, our study aims to describe concurrent use of cigarettes, electronic cigarettes, and waterpipe among Serbian students aged 13–15 years and to explore factors associated with tobacco/nicotine product use across different types of users grouped according to single-, dual- or poly-tobacco use.

## METHODS

This is a secondary analysis of cross-sectional data obtained through the Global Youth Tobacco Survey conducted in Serbia in 2017. A two-stage cluster sample design was used to produce a representative sample of students aged 13–15 years in grades 7 and 8 of Primary school and grade 1 of Secondary school. Methodology and sampling are described in detail elsewhere^19^.

A total of 3861 students completed the questionnaire, of which 3362 were aged 13–15 years. The response rates were 82.9% at the school level, 78.5% at the class level and 80.3% at the student level. The overall response rate was 52.2%.

### Variables

#### Tobacco use

Current users are defined as students that used a product of this study’s interest (cigarette, e-cigarette, waterpipe) at least once in the past 30 days, while ‘concurrent’ users refers to the students that used two or three products within this period. Students were first categorized into the following eight groups: those who do not currently use any tobacco/nicotine product; exclusive cigarette users; exclusive waterpipe users; exclusive e-cigarette users; e-cigarette and waterpipe users who do not smoke cigarettes; cigarette and e-cigarette users; cigarette and waterpipe users; and all three products (cigarettes, e-cigarettes, waterpipe) users. In the next step, users were categorized into the four categories: those who do not currently use any tobacco/nicotine product; exclusive cigarette users; those who use e-cigarettes and/or waterpipes, but not cigarettes; and those who use cigarettes concurrently with either e-cigarettes, waterpipes or both products.

#### Sociodemographic and psychosocial variables

Sociodemographic variables included sex, school grade, and pocket money (average amount of money that students can spend weekly on themselves) as a proxy of socioeconomic status. The age of the students who participated in the study was 13–15 years. The grade was chosen as a demographic variable over the age, as in each grade there is the possibility of finding students of different age. The majority of students aged 13 years was in the 7th grade of Primary school (96%), and also most of those who were 14 years old were in the 8th grade (66.1%) while 63.9% of the students aged 15 years were in the 1st grade of Secondary school.

Smoking frequency among parents, siblings and friends was assessed by the question: ‘How often do you see your father/mother/sibling smoking in your home’, with response options ‘About every day’ or ‘Sometimes’ (recoded into one category), and ‘Never’.

Attitudes toward smoking were assessed with the following set of questions:

- ‘Once someone has started smoking tobacco, do you think it would be difficult for them to quit?’, with response options ‘Definitely not’ or ‘Probably not’ (recoded to No) and ‘Probably yes’ or ‘Definitely yes’ (recoded to Yes);
- ‘Do you think smoking tobacco is harmful to your health?’, with response options ‘Definitely not’ or ‘Probably not’ (recoded to No), ‘Probably yes’ or ‘Definitely yes’ (recoded to Yes);
- ‘Do you think smoking tobacco helps people feel more comfortable or less comfortable at celebrations, parties, or in other social gatherings?’, with response options ‘More comfortable’, ‘Less comfortable’, or ‘No difference whether smoking or not’;
- ‘During the past 30 days, did you see or hear any anti-tobacco messages at sports events, fairs, concerts, or community events, or social gatherings?’ (No, Yes);
- ‘During the past 12 months, were you taught in any of your classes about the dangers of tobacco use?’ (No, Yes);
- ‘During the past 30 days, did you see any advertisements or promotions for tobacco products at points of sale (such as grocery stores, shops, kiosks, etc.)?’ (No, Yes);
- ‘Do you have something (e.g. t-shirt, pen, backpack) with a tobacco product brand logo on it?’ (No, Yes).

### Statistical analysis

Descriptive statistics were used for presenting the frequency of different categories of students according to their nicotine/tobacco product use status. Differences in the prevalence of products were assessed using the χ^2^-test, while in order to evaluate the differences for multiple comparisons we used the z-test with Bonferroni adjustment. Logistic regression was applied for exploring the association of different categories of users with sociodemographic and psychosocial characteristics and reported as adjusted odds ratios (OR) and 95% CI. Level of significance was set at p≤0.05. Data were analyzed using SPSS version 20.

## RESULTS

The majority of the students (81.4%) who participated in this survey declared that they were not using any product in the last 30 days. The prevalence with regard to the product use during the past 30 days seems to be higher for the cigarettes as 11% of the students reported consuming them, followed by waterpipes used by 9% of the students, and e-cigarettes used by 6.2% of the students. The top four patterns of tobacco/nicotine products use are: exclusive cigarette smoking (5.8%), exclusive waterpipe smoking (4.1%), exclusive cigarette and waterpipe use (2.5%), and exclusive e-cigarette use (2.4%). Among students in the 7th grade of Primary school, e-cigarette and/or waterpipe tobacco smoking with no cigarette smoking is threefold higher (7.5%) than exclusive cigarette use (2.5%) (Table 1).

**Table 1:** Prevalence of electronic cigarette, tobacco and waterpipe use and different patterns of use in past 30 days

*Type of products used*	*Total N % (95% CI)*	*Sex N % (95% CI)*		*Grade N % (95% CI)*		

		*Boys*	*Girls*	*7th, Primary*	*8th, Primary*	*1st, Secondary*

*N*	*3256*	*1613*	*1637*	*1218*	*1210*	*815*
**Current prevalence**						
**Cigarette smoking**	362	177	185	57	119	184
	11.0 (9.9–12.1)	10.9 (9.3–12.5)	11.2 (9.6–12.9)	4.6 (3.3–5.9)	9.7 (7.9–11.5)	22.5 (19.3–25.6)
**Waterpipe smoking**	298	152	146	71	96	128
	9.0 (8.0–10.0)	9.2 (7.7–10.7)	8.7 (7.2–10.1)	5.7 (4.3–7.11)	7.7 (6.1–9.3)	15.4 (12.7–18.1)
**E-cigarette use**	206	125	81	70	74	63
	6.2 (5.3–7.1)	7.6 (6.2–9.0)	4.8 (3.7–5.9)	5.6 (4.2–7.0)	5.9 (4.5–7.3)	7.6 (5.6–9.6)
**Prevalence by patterns of use**						
**None of the products**	2652	1306	1341	1070	1013	560
	81.4 (79.9–82.8)	81.0 (78.9–83.1)	81.9 (79.9–84.0)	87.8 (85.8–89.8)	83.7(81.4–85.6)	68.7(65.2–72.2)
**Exclusive cigarette smoking**	190	93	97	30	64	95
	5.8 (4.9–6.6)	5.8 (4.6–7.0)	5.9 (4.7–7.1)	2.5 (1.6–3.4)	5.3 (3.9–6.7)	11.7 (9.3–14.1)
**E-cigarette and/ or waterpipe (no cigarette)**	244	130	114	91	79	73
	7.5 (6.5–8.5)	8.1 (6.7–9.5)	7.0 (5.7–8.3)	7.5 (5.9–9.1)	6.5 (5.0–8.0)	9.0 (6.9–11.1)
*Exclusive waterpipe use*	133	63	70	40	42	50
	4.1 (3.4–4.8)	3.9 (2.9–4.9)	4.3 (3.2–5.4)	3.3 (2.2–4.4)	3.5 (2.4–4.6)	6.1 (4.3–7.9)
*Exclusive electronic cigarette*	79	46	33	39	27	13
	2.4 (1.8–2.9)	2.9 (2.0–3.8)	2.0 (1.3–2.7)	3.2 (2.1–4.3)	2.2 (1.3–3.1)	1.6 (0.7–2.5)
*Electronic cigarettes and waterpipe*	32	21	11	12	10	10
	1.0 (0.6–1.4)	1.3 (0.7–1.9)	0.7 (0.2–1.1)	1.0 (0.4–1.6)	0.8 (0.3–1.3)	1.2 (0.4–2.0)
**Cigarette and other products**	170	84	85	27	54	87
	5.3 (4.5–6.1)	5.3 (4.1–6.5)	5.2 (4.0–6.4)	2.2 (1.3–3.1)	4.5 (3.2–5.8)	10.7 (8.4–13.0)
*Cigarette and electronic cigarettes*	44	22	22	9	15	20
	1.4 (0.9–1.8)	1.4 (0.8–2.0)	1.3 (0.7–1.9)	0.7 (0.2–1.2)	1.2 (0.5–1.9)	2.5 (1.3–3.7)
*Cigarettes and waterpipe*	80	30	50	12	19	48
	2.5 (1.9–3.1)	1.9 (1.2–2.6)	3.1 (2.2–4.0)	1.0 (0.4–1.6)	1.6 (0.6–1.9)	5.9 (4.1–7.7)
*All three products*	46	32	13	6	20	19
	1.4 (0.9–1.8)	2.0 (1.3–2.7)	0.8 (0.3–1.3)	0.5 (0.1–0.9)	1.7 (0.9–2.5)	2.3 (1.2–3.4)

**Figure 1:**
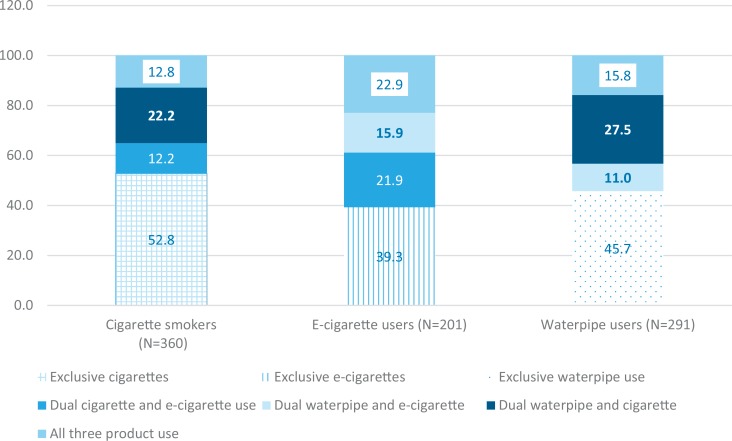
Concurrent use of tobacco/nicotine products among cigarette, waterpipe, and e-cigarette users among Serbian youth

Among current cigarette smokers, more than half were exclusive cigarette smokers (52.8%), while among current e-cigarette users, only 39.3% were exclusive e-cigarette users. Similarly, less than half (45.7%) of current waterpipe tobacco smokers used only this product (Figure 1).

Chi-squared analysis and z-test showed that there were significant differences for all tested variables between abstainers from any tobacco/nicotine product, exclusive cigarette smokers, users of waterpipe and/or e-cigarettes who do not smoke cigarettes, and cigarette and other product users.

Exemptions were found regarding the gender and for noticing anti-tobacco messages on media, while seeing the father smoking at home was close to the p-value threshold for statistical significance (p<0.05) (Table 2).

**Table 2:** Socioeconomic and psychosocial characteristics of tobacco/nicotine users by patterns of use within the past 30 days

*Variables*			*None of the products (N=2652) % (95% CI)*	*Exclusive cigarette smokers (N=190) % (95% CI)*	*Waterpipe and/or e-cigarette users (N=244) % (95% CI)*	*Cigarette and other product users (N=170) % (95% CI)*
	Total (3861)		81.4 (80.1–82.7)	5.8 (5.0–6.6)	7.5 (6.6–8.4)	5.3 (4.6–6.0)
Gender χ^2^=1.425, p=0.700	Male	A	81.0 (78.9–83.1)	5.8 (4.6–7.0)	8.1 (6.7–9.5)	5.2 (4.0–6.4)
	Female	B	81.9 (78.9–83.9)	5.9 (4.7–7.1)	7.0 (5.7–8.3)	5.2 (4.0–6.4)
School grade χ^2^=168.063, p=0.000	7th, Primary	A	87.8 (85.8–89.9) BC	2.5 (1.6–3.4)	7.5 (5.9–9.1)	2.2 (1.3–3.1)
	8th, Primary	B	83.7 (81.4–86.0) C	5.3 (3.9–6.7) A	6.5 (5.0–8.0)	4.5 (3.2–5.8) A
	1st, Secondary	C	68.7 (65.2–72.2)	11.7 (9.3–14.1) AB	9.0 (6.9–11.1)	10.7 (8.4–13.0) AB
Pocket money χ^2^=141.591, p=0.000	no pocket money	A	87.8 (83.1–92.5) C	2.7 (0.4–5.0)	7.2 (3.5–10.9)	2.3 (0.1–2.5)
	≤1000	B	85.8 (84.2–87.4) C	5.0 (4.0–6.0)	5.6 (4.6–6.6)	3.6 (2.8–4.4)
	>1000	C	68.0 (64.5–71.5)	9.1 (7.0–11.2) AB	12.7 (10.2–15.2) B	10.3 (8.0–12.6) AB
Father smoking at home χ^2^=6.165, p=0.049	Never	A	83.2 (81.1–85.3) B	5.5 (4.2–6.8)	6.9 (5.5–8.3)	4.3 (3.2–5.4)
	Sometimes/about every day	B	79.9 (77.7–82.1)	6.0 (4.7–7.3)	8.2 (6.7–9.7)	5.8 (4.5–7.1)
Mothers smoking at home χ^2^=18.112, p=0.000	Never	A	83.6 (81.7–85.5) B	5.5 (4.3–6.7)	7.0 (5.7–8.3)	3.8 (2.8–4.8)
	Sometimes/about every day	B	78.4 (76.0–80.8)	6.7 (5.3–8.1)	8.1 (6.5–9.7)	6.7 (5.3–8.1) A
Siblings smoking at home χ^2^=72.646, p=0.000	Never	A	83.8 (82.2–85.4) B	5.0 (4.0–6.0)	7.3 (6.2–8.4)	3.8 (3.0–4.6)
	Sometimes/about every day	B	68.8 (64.1–73.5)	11.4 (8.2–14.6) A	9.1 (6.2–12.0)	10.7 (7.6–13.8) A
Number of smokers among closest friends χ^2^=696.254, p=0.000	None of them	A	92.6 (91.2–94.0) BC	0.7 (0.2–1.2)	6.0 (4.7–7.3)	0.7 (0.2–1.2)
	Some of them	B	81.8 (79.4–94.0) C	5.7 (4.3–7.1) A	8.2 (6.5–7.3)	4.2 (3.0–5.4) A
	Majority (about half and more of them)	C	49.2 (44.6–53.8)	20.9 (17.1–24.7) AB	9.9 (7.1–12.7) A	20.0 (16.3–23.7) AB
Think tobacco is harmful to their health χ^2^=73.042, p=0.000	Yes	A	83.0 (81.6–84.4) B	5.8 (4.9–6.7)	6.6 (5.6–7.6)	4.6 (3.8–5.4)
	No	B	63.6 (57.1–70.1)	6.8 (3.4–10.2)	18.0 (12.8–23.2) A	11.6 (7.2–16.0) A
Smoking helps feel comfortable at social events χ^2^=31.788, p=0.000	No difference	A	84.6 (82.1–87.1) C	4.7 (3.2–6.2)	6.4 (4.7–8.1)	4.3 (2.9–5.7)
	Less comfortable	B	84.9 (81.6–88.2) C	3.6 (1.9–5.3)	8.7 (6.1–11.3)	2.7 (1.2–4.2)
	More comfortable	C	78.6 (76.5–80.7)	7.3 (6.0–8.6) AB	7.7 (6.3–9.1)	6.3 (5.0–7.6) B
Hard to quit once someone starts smoking χ^2^=50.630, p=0.000	Yes	A	83.9 (82.4–85.4) B	5.2 (4.3–6.1)	7.1 (6.0–8.2)	3.9 (3.1–4.7)
	No	B	73.7 (70.3–77.1)	8.0 (5.9–10.1) A	9.0 (6.8–11.2)	9.3 (7.0–11.6) A
Saw anti-tobacco message χ^2^=2.294, p=0.514	Yes	A	81.4 (79.3–83.5)	6.4 (5.1–7.7)	7.1 (5.7–8.5)	5.1 (3.9–6.3)
	No	B	82.0 (80.0–84.0)	5.3 (4.4–6.5)	7.6 (6.2–9.0)	5.1 (4.0–6.2)
Being taught in school about harmful effects of smoking χ^2^=8.612, p=0.035	Yes	A	82.7 (81.0–84.4) B	5.9 (4.8–7.0)	7.0 (5.8–8.2)	4.4 (3.4–5.4)
	No	B	79.5 (77.0–82.0)	5.7 (4.3–7.1)	8.5 (6.8–10.2)	6.3 (3.9–6.7) A
Exposed to point of sale marketing χ^2^=87.943, p=0.000	No	A	86.5 (84.8–88.2) B	4.3 (3.3–5.3)	6.3 (5.1–7.5)	2.9 (2.1–3.7)
	Yes	B	74.3 (71.7–76.9)	7.9 (6.3–9.5) A	9.2 (7.5–10.9) A	8.6 (7.0–10.2) A
Having tobacco industry item χ^2^=145.474, p=0.000	No	A	84.5 (83.1–85.9) B	5.0 (4.1–5.9)	6.6 (5.6–7.6)	3.8 (3.0–4.6)
	Yes	B	60.1 (54.5–65.7)	12.0 (8.3–15.7) A	12.5 (8.7–16.3) A	15.4 (11.3–19.5) A

For each pair of smoking categories, proportions (for each row) are compared using a z-test with significance level at 0.05. If a pair of values is significantly different, the values have different letters assigned to them.

The results of the logistic regression show that students in the 1st grade of Secondary school compared to students from the 7th grade of Primary school have 3 times greater possibility of being exclusive cigarette smokers and almost 5 times higher odds of being users of cigarettes and other products, compared to not any tobacco/nicotine users. The grade was not found to be a significant predictor of e-cigarette and/or waterpipe users who do not smoke cigarettes. The odds of being tobacco/nicotine user increase with the amount of available pocket money. However, with regard to the pocket money, a statistically significant difference was found only in the model that compared waterpipe and/ or e-cigarette (no cigarette) users, indicating that students with the highest amount of pocket money have 3 times higher risk of being waterpipe and/or e-cigarette users compared to those who do not use any tobacco/nicotine product.

Logistic regression analysis, also, shows that students who consider tobacco as not harmful to health have 2.9 higher odds of being electronic cigarette and/or waterpipe users, as well as cigarette and other emerging products users.

The highest odds of being either exclusive cigarette smokers, electronic cigarette and waterpipe users and users who smoke cigarettes and other products compared to the students who do not use any of these products, are students who report that the majority of their friends smoke. Another predictor, found to be statistically significant for the explored tobacco/nicotine user groups, is exposure to point-of-sale marketing (Table 3).

**Table 3:** Logistic regression results of the association between sociodemographic and psychosocial characteristics with different patterns of nicotine/tobacco use

*Variables*		*Exclusive cigarette vs not any product use OR (95% CI)*	*Waterpipe and or/e-cigarette use vs not any product use OR (95% CI)*	*Cigarette and other product users vs not any product use OR (95% CI)*
Gender	Male (ref)			
	Female	0.97 (0.64–1.47)	0.99 (0.71–1.38)	0.99 (0.60–1.65)
School grade	7th, Primary			
	8th, Primary	1.64 (0.88–3.06)	0.72 (0.48–1.07)	1.69 (0.73–3.89)
	1st, Secondary	3.06 (1.64–5.74)**	0.88 (0.56–1.39)	4.88 (2.15–11.07)**
Pocket money	No pocket money (ref)			
	≤1000 RSD	1.72 (0.48–6.14)	0.88 (0.43–1.83)	2.62 (0.32–21.10)
	>1000 RSD	2.39 (0.66–8.68)	2.34 (1.12–4.91)*	5.92 (0.73–48.17)
Father smoking at home	Never(ref)			
	Sometimes/about every day	1.03 (0.67–1.58)	1.39 (0.98–1.98)	0.77 (0.45–1.33)
Mother smoking at home	Never(ref)			
	Sometimes/about every day	1.04 (0.68–1.60)	1.01 (0.71–1.44)	1.57 (0.91–2.72)
Siblings smoking at home	Never(ref)			
	Sometimes/about every day	2.45 (1.54–3.87)**	0.88 (0.55–1.44)	2.31 (1.30–4.12)*
Number of smokers among closest friends	None (ref)			
	Some	5.98 (2.62–13.64)**	1.76 (1.20–2.59)*	11.6 (2.71–49.66)**
	Majority (about half and more of them)	33.2 (14.52–75.90)**	2.57 (1.56 –4.25)**	52.37 (12.28–223.223)**
Think tobacco is harmful to their health	Yes (ref)			
	No	1.10 (0.49–2.47)	2.90 (1.61–5.24)**	2.90 (1.29–6.52)*
Smoking helps feel comfortable at social events	No difference (ref)			
	Less comfortable	1.68 (0.83–3.42)	1.36 (0.81–2.29)	1.21 (0.45–3.27)
	More comfortable	1.74 (1.08–2.82)*	1.34 (0.91–1.97)	1.74 (0.96–3.16)
Hard to quit once someone starts smoking	Yes (ref)			
	No	1.65 (1.03–2.64)*	0.83 (0.54–1.29)	2.25 (1.29–3.92)*
Saw anti-tobacco message	Yes (ref)			
	No	0.72 (0.48–1.07)	1.16 (0.83–1.62)	0.87 (0.52–1.45)
Being taught in school about harmful effects of smoking	Yes (ref)			
	No	0.97 (0.63–1.48)	1.23 (0.87–1.72)	0.94 (0.56–1.57)
Exposed to point of sale marketing	No (ref)			
	sale marketing	1.82 (1.22–2.73)*	1.64 (1.18–2.28)*	3.40 (1.99–5.80)**
Having tobacco industry item	No (ref)			
		1.59 (0.93–2.74)	2.03 (1.30–3.19)*	2.80 (1.58–4.96)**

* p<0.05

** p<0.001.

RSD: Serbian dinar, exchange rate 1000 RSD about 9.42 US$.

## DISCUSSION

Almost one-fifth (18.6%) of the students aged 13–15 years who participated in this survey in Serbia smoke cigarettes or use any of the products (cigarette, e-cigarette, waterpipe) that were explored in this study.

High prevalence of waterpipe tobacco smoking needs great attention, given the fact that specialized waterpipe cafes have appeared in Serbia a few years ago and are spreading rapidly since then. With these findings, Serbia joins the group of Eastern Mediterranean and Eastern European countries such as Lebanon, Latvia, Czech Republic and Estonia that also have a high prevalence of waterpipe tobacco smoking^14^.

The increasing e-cigarette use is also of great concern. This product has been present in the Serbian market for more than 10 years. However, only in 2016 has the ban on advertising of electronic cigarettes been introduced and that might have prevented a faster increase in the prevalence of electronic cigarette use among youth, since according to the literature there is an association between e-cigarette marketing and e-cigarette use among adolescents^20^. In the previous 2013 Global Youth Tobacco Survey, the question on electronic cigarette use was not included, which prevented us from observing the trend.

An additional finding was that slightly more than half of cigarette smokers are exclusive users, while among e-cigarette users and waterpipe users the exclusive use of these products is even lower (39.3% and 45.7%, respectively). Findings are in line with other studies stressing the problem of dual- and poly-tobacco use such as cigarettes and smokeless tobacco^21^, cigarettes and waterpipes^22^, cigarettes and electronic cigarettes^23^, as well as waterpipes, electronic cigarettes and cigarettes^24^.

We too found that there are both similarities and differences among exclusive cigarette users, users of e-cigarettes and/or waterpipe who do not smoke cigarettes and those who use cigarettes and other products. High percentage of e-cigarette use, either exclusively or with waterpipe, might be attributed to curiosity and other factors and needs attention, as it has been shown that there is an association between e-cigarette use and subsequent cigarette smoking initiation^25^.

No gender differences were found across the four categories of students with regard to tobacco/nicotine use (not any tobacco/nicotine product users, exclusive cigarette smokers, waterpipe and/or e-cigarette users who do not smoke cigarettes, and cigarette and other products users). Findings from this study also indicate that exclusive use of cigarettes and use of cigarettes with other products is more common among older students, while differences by school grade are not significant among electronic cigarette and/or waterpipe users who do not smoke cigarettes.

Another finding of ours was that the number of closest friends who smoke was associated with exclusive cigarette smoking, exclusive waterpipe and/or e-cigarette use, and cigarette and other products use. However, parents’ smoking did not influence tobacco/e-cigarette use. These results could be interpreted in the light of high social acceptance of smoking in Serbia, which is, among others, documented by data showing a very low percentage of totally smoke-free households in Serbia (8% in 2018)^26^. Research by Szabo et al.^27^ shows that a home smoking ban is associated with lower likelihood of adolescents experimenting with tobacco and that parents can reduce the influence of friends’ smoking on the smoking behavior of their children^27^. From that perspective, interventions aimed at establishing smoke-free households can help in reducing the prevalence of single- and poly-tobacco use. Parents can prevent children’s smoking through high-quality communication, avoiding negative reactions or punishments, and setting comprehensive smoking rules at home^28^. Therefore, encouraging parents to discuss smoking-related issues regardless of their smoking status is strongly recommended for further research as a promising strategy^29,30^. The association between the approval of using e-cigarettes and cigarettes among friends and family and cigarette and e-cigarette use^31^ highlights the need to focus on preventive interventions not only addressed to students but also to those people significant to them.

Waterpipe and e-cigarette use was also found to be associated with psychosocial factors, such as higher pocket money, grade, but also exposure to tobacco industry advertising. Similar to our findings, other research has shown an association of poly-tobacco use with peer influence and exposure to tobacco industry tactics^32^.

Our results show that among the students who took this survey in Serbia, those who consider tobacco use as less harmful have higher odds of being dual- and poly-tobacco/nicotine users. Low-risk perception is found to be associated with e-cigarette use^33^ and waterpipe tobacco smoking^34,35^ in other studies also. However, we found that tobacco harm perception was a significant predictor of dual-tobacco users relative to single-product users, as reported in a study in the USA^36^.

It should be kept in mind that many long-term health consequences of tobacco use could develop in the future, while adolescents mostly focus on the short-term rewards. However, focusing only on the risks might make the smoking habit more attractive to youth. Failing to acknowledge this and focusing only on future risks may lead to losing the opportunity to reach adolescents with tailored interventions such as emphasizing the perceived benefits of quitting in the present^37^. Therefore, as recommended by other studies, the role of perceived social benefits should be considered in such interventions, as well as the need to increase adolescents’ awareness of the addictive nature of nicotine^38^.

Comparability of our findings with other studies is limited as there are not many studies exploring different categories of smokers based on data from 2017 onward and collected from youth aged 13–15 years. This is of importance due to the rapid change in tobacco product use, which shows the growing popularity of waterpipe tobacco smoking and e-cigarettes in many countries. Data are exceptionally scarce in developing countries that are facing similar challenges, such as the Western Balkan countries. Some results from our study deserve special attention from the perspective of interventions such as those showing that among 7th grade students who are mostly 13 years old, the prevalence of dual waterpipe and e/cigarette use is much higher than exclusive cigarette smoking (2.2% vs 7.5%). Despite the fact that dual- and poly-tobacco/nicotine product use is of growing concern, research points to limited evidence on effective interventions aimed at dual- and poly-tobacco use^39^. As we could not identify, neither in Serbia nor in the Western Balkan region, any data on concurrent use of cigarettes, waterpipes and electronic cigarettes that could guide further interventions, we believe that both policymakers and experts could benefit from our findings.

### Limitations

The well-developed and elaborated methodology of the Global Youth Tobacco survey was applied for the collection of data. One of the limitations is the overall response rate, which was less than 60%; thus, results are not representative of the Serbian student population. Other limitations include recall bias (minimal, given the fact that we analyzed behavior that happened in the last 30 days), bias related to self-reported smoking behavior without biochemical verification, as well as faking good or bad phenomena. In addition, we were not able to make a distinction between electronic cigarette with and without nicotine, or between waterpipes with tobacco and herbal or other mixtures.

## CONCLUSIONS

Dual- and poly-tobacco use indicates the need for tobacco control initiatives that will address these challenges. The higher prevalence of waterpipe and e-cigarette use than cigarette use among younger students highlights the changing patterns of nicotine exposure and need for action. The mutual predictors for all explored patterns of tobacco/nicotine tobacco users were: smoking of the closest friends, and exposure to point-of-sale marketing. These findings are a clear indication of what should be addressed in further interventions aimed at youth, both in terms of legislation and its enforcement and other interventions such as campaigns and programs in different settings such as school and home.
